# Supplementary material for: Predictors of in-hospital mortality among cirrhotic patients in Ethiopia: A multicenter retrospective study
Source: PLoS One. 2025 Apr 29;20(4):e0322532. doi: 10.1371/journal.pone.0322532 (PMC12040200; doi:10.1371/journal.pone.0322532)
Supplement: Attachment — Submitted filename: Cirrhosis Response to reviewers.docx [file pone.0322532.s004.docx]

PONE-D-24-44329

Predictors of In-hospital Mortality among Cirrhotic Patients in Ethiopia: A Multicenter Retrospective Study

*Dear Editor and Reviewers;*

*I hope this letter finds you well. I would like to express my gratitude for taking the time to review our manuscript. Your constructive feedback and comments have been invaluable in improving the quality and clarity of our work.*

*I would like to address the concerns and comments raised in your review. I have carefully considered each one of them and have made the necessary changes to the manuscript accordingly.*

*So please find the following comments and responses.*

Journal Requirements:

When submitting your revision, we need you to address these additional requirements.

1. Please ensure that your manuscript meets PLOS ONE's style requirements, including those for file naming.

- *I have revised the manuscript according to PLOS ONE’s style requirements.*

Reviewers' comments:

Reviewer's Responses to Questions

**Comments to the Author**

1. Is the manuscript technically sound, and do the data support the conclusions?

The manuscript must describe a technically sound piece of scientific research with data that supports the conclusions. Experiments must have been conducted rigorously, with appropriate controls, replication, and sample sizes. The conclusions must be drawn appropriately based on the data presented.

Reviewer #1: Yes

Reviewer #2: Yes

Reviewer #3: Yes

Reviewer #4: Partly

2. Has the statistical analysis been performed appropriately and rigorously?

Reviewer #1: Yes

Reviewer #2: Yes

Reviewer #3: Yes

Reviewer #4: Yes

3. Have the authors made all data underlying the findings in their manuscript fully available?

The [PLOS Data policy](http://track.editorialmanager.com/CL0/http:%2F%2Fwww.plosone.org%2Fstatic%2Fpolicies.action%23sharing/1/010f01942b9a55ca-b6faa258-6ccd-4eca-a986-c856ef0f44b8-000000/cpTDsSoXG8X_M4ZdVXOGex3IXO8jBGDXdn1j0xeitAU=192) requires authors to make all data underlying the findings described in their manuscript fully available without restriction, with rare exception (please refer to the Data Availability Statement in the manuscript PDF file). The data should be provided as part of the manuscript or its supporting information, or deposited to a public repository. For example, in addition to summary statistics, the data points behind means, medians and variance measures should be available. If there are restrictions on publicly sharing data—e.g. participant privacy or use of data from a third party—those must be specified.

Reviewer #1: Yes

Reviewer #2: Yes

Reviewer #3: Yes

Reviewer #4: Yes

4. Is the manuscript presented in an intelligible fashion and written in standard English?

PLOS ONE does not copyedit accepted manuscripts, so the language in submitted articles must be clear, correct, and unambiguous. Any typographical or grammatical errors should be corrected at revision, so please note any specific errors here.

Reviewer #1: Yes

Reviewer #2: Yes

Reviewer #3: No

Reviewer #4: Yes

- *Thank you for the positive response. Some sections of the manuscript are now edited to make it clearer.*

5. Review Comments to the Author

Reviewer #1: This is to determine predictors of In-hospital Mortality among Cirrhotic Patients in Ethiopia, a developing country. The manuscript is well written

- *Thank you for the positive response.*

There are few issues the authors have to address

1. This could be because of the high viral hepatitis burden (second paragraph the reason for younger age of diagnosing liver cirrhosis..................... not only high burden but early age of acquisition of these viruses

- *Thanks for the insight. It is now included in the manuscript.*

2. The higher prevalence of liver cirrhosis and its complications in men is likely due to a combination of behavioral factors such as alcohol consumption, and biological factors such as lower levels of estrogen, which have been shown to have anti-fibrotic and anti-inflammatory effects on the liver that collectively increase their susceptibility to the development and progression of various liver diseases leading to cirrhosis ...................... this sentence is too long and therefore not clear

- *The sentence is now rewritten in a clear & concise manner.*

3. Kindly compare and contrast the percentage of ascites recorded in your study in the discussion section

- *The comparison of the prevalence of ascites in our study population with previous studies is now incorporated in the manuscript.*

4. Am not seeing authors contribution in the manuscript

- *Sorry for that. Authors contribution is now included in the revised manuscript.*

5. Kindly check and rectify some of the reference

- *Thanks for the feedback. The reference section is now edited.*

Reviewer #2: Thank you, dear authors, for this interesting manuscript.

- *Thank you for taking your time to review our manuscript.*

Here are my comments:

#Introduction: At the end of the introduction, please explicitly state that this manuscript has been reported in accordance with the STROBE guidelines. Cite the relevant reference and include the checklist at the end of the manuscript.

- *Statement of Reporting Compliance with the STROBE guidelines along with the reference is included at the end of the introduction. The checklist is also included with manuscript submission as a supplementary material.*

#Kindly provide the reference for the diagnostic criteria of cirrhosis mentioned in the study

- *The reference for the diagnostic criteria of cirrhosis used in the study is included in the revised manuscript.*

#The manuscript requires minor grammatical corrections throughout to enhance clarity and readability.

- *Thank you for the feedback. The manuscript is now edited for language usage, spelling, and grammar.*

Reviewer #3: Cirrhosis is a common disease, and as a multicenter study, 299 cases is not a lot actually. There have been many previous studies on the etiology, but the correlation analysis of related factors for death in patients with liver cirrhosis in Ethiopia is relatively few, so it has certain novelty and clinical value. This study calls attention to certain risk factors and take earlier prevention measures.

- *Thank you for the positive response.*

However, there are many defects in the manuscript that need to be modified:

1.Professional language polishing is needed. There are too many inappropriate spellings (including the Abstract) in the whole article, such as the first letter of many words in the sentence should be lowercase; abbreviations and full names of terms cross over and over again in the article; incorrect use of symbols.

- *The manuscript is now edited for language usage, spelling, and grammar.*

2.In the paragraph 3 of Introduction, autoimmune liver disease should be also an important cause.

- *Thank you for the reminder. Autoimmune hepatitis is now included as one of the major causes of liver disease.*

3. In the Methodology, the diagnostic criteria for the compensatory and decompensated stage of cirrhosis are quite different and should be distinguished.

- *We totally agree with the reviewer's comment and have included information about liver decompensation in the second paragraph of the introduction. The reason we didn’t include it in the methodology section is because the current study included all patients with cirrhosis despite their compensation status.*

4. The Child-Pugh and MELD Na scoring criteria need to be briefly mentioned in the Methodology.

- *The Child-Pugh and MELD Na scoring criteria are briefly discussed in the methodology (data collection tools and procedures) section of the revised manuscript.*

5. The abbreviations of the terms in the Table need to be annotated.

- *Thank you for your suggestion. A list of abbreviations with their full forms has been added at the base of the table to enhance clarity and readability.*

6. What does COR refer to in Table2?

- *COR in table 2 refers to crude odds ratio. Now, the abbreviation with its full form is included at the base of the table.*

7. More discussion of hepatic encephalopathy is recommended.

- *Thank you for the input. In the revised manuscript, discussion about the spectrum, prevalence, and classification of hepatic encephalopathy is included.*

8. Measures to prevent risk factors for death might be appropriately mentioned in the Discussion.

- *Measures to prevent risk factors for in-hospital mortality has now been included in the discussion section of the revised manuscript.*

Reviewer #4: Section Operational definition
1. Line 124:
Liver cirrhosis: was diagnosed based on the presence of two or more of the following
It would be more appropriate to write
Liver cirrhosis was diagnosed based on the presence of two or more of the following :

- *Thank you for the suggestion. Correction has been made accordingly.*

2. Section Conclusion
Do you think that a study carried out in 3 hospitals out of several others is enough to conclude about the whole country?

- *We acknowledge that including additional hospitals could enhance the generalizability of the findings, but the three hospitals included in the study are the largest referral centers in the country, which receive a high volume of patients with cirrhosis from various regions, and we believe the findings are representative of the broader national population.*
