# Supplementary material for: Predictors of in-hospital mortality among cirrhotic patients in Ethiopia: A multicenter retrospective study
Source: PLoS One. 2025 Apr 29;20(4):e0322532. doi: 10.1371/journal.pone.0322532 (PMC12040200; doi:10.1371/journal.pone.0322532)
Supplement: Attachment — Submitted filename: Review comments.docx [file pone.0322532.s003.docx]

1. Please highlight or red the modified areas, otherwise it will increase the difficulty of reviewing again.
2. Although the language is much better than before, there are still some minor flaws, such as Child-Pugh is sometimes capitalized and sometimes lowercase, Median Age in Years maybe “Median age in years”？
3. What COR is in Table 2 remains unexplained.

4. Please explain why cirrhosis cannot be distinguished and analysed according to decompensated and decompensated periods?
