# Supplementary material for: Predictors of in-hospital mortality among cirrhotic patients in Ethiopia: A multicenter retrospective study
Source: PLoS One. 2025 Apr 29;20(4):e0322532. doi: 10.1371/journal.pone.0322532 (PMC12040200; doi:10.1371/journal.pone.0322532)
Supplement: Attachment — Submitted filename: Response to Reviewers.docx [file pone.0322532.s005.docx]

PONE-D-24-44329R1

Predictors of In-hospital Mortality among Cirrhotic Patients in Ethiopia: A Multicenter Retrospective Study

*Dear Editor and Reviewers;*

*I hope this letter finds you well. I would like to express my gratitude for taking the time to review our manuscript. Your constructive feedback and comments have been invaluable in improving the quality and clarity of our work.*

*I would like to address the concerns and comments raised in your review. I have carefully considered each one of them and have made the necessary changes to the manuscript accordingly.*

*So please find the following comments and responses.*

Journal Requirements:

Please review your reference list to ensure that it is complete and correct. If you have cited papers that have been retracted, please include the rationale for doing so in the manuscript text, or remove these references and replace them with relevant current references. Any changes to the reference list should be mentioned in the rebuttal letter that accompanies your revised manuscript. If you need to cite a retracted article, indicate the article’s retracted status in the References list and also include a citation and full reference for the retraction notice.

- *I have reviewed the reference list and found it to be complete, and no retracted paper was cited.*

Reviewers' comments:

Reviewer's Responses to Questions

**Comments to the Author**

1. If the authors have adequately addressed your comments raised in a previous round of review and you feel that this manuscript is now acceptable for publication, you may indicate that here to bypass the “Comments to the Author” section, enter your conflict of interest statement in the “Confidential to Editor” section, and submit your "Accept" recommendation.

Reviewer #1: All comments have been addressed

Reviewer #3: (No Response)

- *Thank you for the positive response.*

2. Is the manuscript technically sound, and do the data support the conclusions?

   The manuscript must describe a technically sound piece of scientific research with data that supports the conclusions. Experiments must have been conducted rigorously, with appropriate controls, replication, and sample sizes. The conclusions must be drawn appropriately based on the data presented.

Reviewer #1: Yes

Reviewer #3: Yes

- *Thank you for the positive response.*

3. Has the statistical analysis been performed appropriately and rigorously?

Reviewer #1: Yes

Reviewer #3: Yes

- *Thank you for the positive response.*

4. Have the authors made all data underlying the findings in their manuscript fully available?

   The [PLOS Data policy](http://track.editorialmanager.com/CL0/http:%2F%2Fwww.plosone.org%2Fstatic%2Fpolicies.action%23sharing/1/010f01958c73c22c-f0a80b5f-4afc-4bfa-809a-b2bfe2d14b3b-000000/Sklc3mQvTbDiW7IMB6IysmbJ60T7zLneyVOSS91mLdw=202) requires authors to make all data underlying the findings described in their manuscript fully available without restriction, with rare exception (please refer to the Data Availability Statement in the manuscript PDF file). The data should be provided as part of the manuscript or its supporting information, or deposited to a public repository. For example, in addition to summary statistics, the data points behind means, medians and variance measures should be available. If there are restrictions on publicly sharing data—e.g. participant privacy or use of data from a third party—those must be specified.

Reviewer #1: Yes

Reviewer #3: Yes

- *Thank you for the positive response.*

5. Is the manuscript presented in an intelligible fashion and written in standard English?

   PLOS ONE does not copyedit accepted manuscripts, so the language in submitted articles must be clear, correct, and unambiguous. Any typographical or grammatical errors should be corrected at revision, so please note any specific errors here.

Reviewer #1: Yes

Reviewer #3: Yes

- *Thank you for the positive response.*

6. Review Comments to the Author

   Please use the space provided to explain your answers to the questions above. You may also include additional comments for the author, including concerns about dual publication, research ethics, or publication ethics. (Please upload your review as an attachment if it exceeds 20,000 characters)

Reviewer #1: All my previous recommendations have been addressed. However, the first paragraph of the results section should be under the methods section, maybe at the sample size. The reasons should be giving for those not meeting the inclusion criteria.

- *Thank you for the suggestion. The first part of the result section is now included in the sample size section of the methodology part, and the explanation for being excluded from the study are also explained.*

Reviewer #3:

1. Please highlight or red the modified areas, otherwise it will increase the difficulty of reviewing again.

- *I apologize for any trouble this may have caused. The marked-up copy of our manuscript that highlights changes made to the original version is uploaded as a separate file labeled 'Revised Manuscript with Track Changes,' and an unmarked version of our revised paper without tracked changes is uploaded as a separate file labeled 'Manuscript.'.*

2. Although the language is much better than before, there are still some minor flaws, such as Child-Pugh is sometimes capitalized and sometimes lowercase, Median Age in Years maybe “Median age in years”？

- *Thank you for the feedback. The aforementioned capitalization mistakes are now corrected.*

3. What COR is in Table 2 remains unexplained.

- *COR and AOR are now described as crude odds ratio and adjusted odds ratio at the bottom of the table.*

4. Please explain why cirrhosis cannot be distinguished and analyzed according to decompensated and decompensated periods?

- *Thank you for raising the idea. Analyzing patients survival based on their compensation status is advisable. However, the vast majority of our patients were admitted due to upper GI bleeding, hepatic encephalopathy, or ascites which are the signs of hepatic decompensation.*
