# Supplementary material for: Predictors of in-hospital mortality among cirrhotic patients in Ethiopia: A multicenter retrospective study
Source: PLoS One. 2025 Apr 29;20(4):e0322532. doi: 10.1371/journal.pone.0322532 (PMC12040200; doi:10.1371/journal.pone.0322532)
Supplement: Attachment — Submitted filename: Reviewer comments.docx [file pone.0322532.s001.docx]

Cirrhosis is a common disease, and as a multicenter study, 299 cases is not a lot actually. There have been many previous studies on the etiology, but the correlation analysis of related factors for death in patients with liver cirrhosis in Ethiopia is relatively few, so it has certain novelty and clinical value. This study calls attention to certain risk factors and take earlier prevention measures.

However, there are many defects in the manuscript that need to be modified:

1. Professional language polishing is needed. There are too many inappropriate spellings (including the Abstract) in the whole article, such as the first letter of many words in the sentence should be lowercase; abbreviations and full names of terms cross over and over again in the article; incorrect use of symbols.
2. In the paragraph 3 of Introduction, autoimmune liver disease should be also an important cause.

3. In the Methodology, the diagnostic criteria for the compensatory and decompensated stage of cirrhosis are quite different and should be distinguished.

4. The Child-Pugh and MELD Na scoring criteria need to be briefly mentioned in the Methodology.

5. The abbreviations of the terms in the Table need to be annotated.

6. What does COR refer to in Table2?

7. More discussion of hepatic encephalopathy is recommended.

8. Measures to prevent risk factors for death might be appropriately mentioned in the Discussion.
